# Large‐Scale Structural Dynamics in the Tail Fiber Modulate the Infective Transition of the T7 Bacteriophage

**DOI:** 10.1002/smll.74269

**Published:** 2026-06-19

**Authors:** Luca Elizabet Kosik, Miklós Cervenak, Dominik Sziklai, Andrea Balogh‐Molnár, Negar Rahimi, Bence Fehér, Soma Yamamoto, Hiroki Konno, Noriyuki Kodera, Holger Flechsig, Romain Amyot, Heinz Amenitsch, Hedvig Tordai, Levente Herényi, Ana Cuervo, Miklós Kellermayer, Bálint Kiss

**Affiliations:** ^1^ Department of Biophysics and Radiation Biology Semmelweis University Budapest Hungary; ^2^ HUN‐REN‐SE Biophysical Virology Group Budapest Hungary; ^3^ Nanobiophysics Research Group HUN‐REN Office for Supported Research Groups Budapest Hungary; ^4^ Graduate School of Natural Science and Technology Kanazawa University Kanazawa Ishikawa Japan; ^5^ WISE Program for Nano‐Precision Medicine, Science, and Technology Kanazawa University Kanazawa Ishikawa Japan; ^6^ WPI Nano Life Science Institute (WPI‐NanoLSI) Kanazawa University Kanazawa Ishikawa Japan; ^7^ Institute of Inorganic Chemistry Graz University of Technology Graz Austria; ^8^ Centro Nacional de Biotecnología (CNB‐CSIC) Madrid Spain

**Keywords:** host‐cell recognition, HS‐AFM, MD simulation, SAXS, single‐particle biophysics, T7 bacteriophage, virus infection

## Abstract

Bacteriophages are gaining interest due to their potential use against multidrug‐resistant bacteria. Here, we investigated the structural dynamics of T7 bacteriophage tail fibers. The T7 virion comprises an icosahedral protein shell and a tail‐fiber complex, which is involved in bacterial target recognition and viral DNA injection. The virus has six L‐shaped, ∼40‐nm‐long fibers connected to the tail‐tube, which are thought to be essential for initial host recognition and, possibly, for surface exploration. By using high‐speed atomic force microscopy (HS‐AFM) and molecular dynamics (MD) simulations combined with small angle X‐ray scattering (SAXS), we analyzed the molecular structure and movements of isolated tail fibers and tail‐fiber complexes. We found that the kink region separating the proximal and distal sections of the fiber acts as a molecular hinge, which allows large‐scale dynamic bending. Furthermore, partial unwinding‐rewinding in the triple‐helical coiled‐coil structure of the proximal section permits gross fiber rotation and twisting. The two dynamic regions allow for large‐scale, rapid fiber flexing and extension, thus enabling an efficient topological search for anchorage sites by the phage on the host surface. Since the fiber‐assisted topological search is likely a universal mechanism of host recognition, modulating it could be used for fine‐tuning the phage‐bacterium infection process.

## Graphical abstract

During host recognition, the fibers of T7 bacteriophages transition from a capsid bound state to an extended conformation, which requires some level of fiber flexibility. By using high‐speed atomic force microscopy, we show that the fibers have an internal molecular hinge and a torsionally compliant coiled‐coil region. We propose that this flexibility is utilized in host surface exploration by walking.

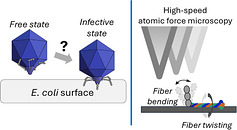

## 1 Introduction

The incidence of infections by multidrug‐resistant bacteria is sharply rising, posing ever more increasing pressure on our healthcare systems to find alternatives to antibiotics [1]. Bacteriophages have emerged as an effective alternative, because they infect bacteria with unparalleled specificity [2, 3, 4]. However, the efficient use of phages as therapeutic agents requires precise knowledge of the molecular mechanisms of their infection cycle, from target recognition to host lysis [5, 6]. Because the infection cycle involves mechanical steps, bulk experiments fail to unravel all mechanistic details. By contrast, single particle approaches shed light on the individual steps of target recognition and infection [2]. A workhorse of virus research, the T7 bacteriophage has long been in the center of focus, yet the exact process of its target recognition and infection mechanisms remained unclear [7]. T7 comprises an icosahedral capsid that encompasses the viral genome, and a tail fiber complex, which is used for host (*E. coli*) recognition and genome delivery [8, 9]. The tail‐fiber complex is attached to a unique capsid vertex through the dodecameric portal protein gp8 [10]. Together, the portal protein, tail tube, and six tail fibers form a portal‐tail complex with a total molecular weight of approximately 1.5 MDa [11]. The tube is formed by proteins gp11 and gp12 [11], with gp11 acting as an adapter between the portal and gp12, and the latter functioning as a gatekeeper that retains the viral DNA. Tail fibers are trimers of the gp17 protein (3 × 62 kDa) that connect to the tail tube via their N‐termini between the dodecameric gp11 and hexameric gp12 rings. The fibers possess an overall L‐shaped structure. The two straight parts of the L, separated by a kink region, are called proximal and distal sections with respect to their position to the tail tube [12]. Most of the distal section's structure has been resolved by X‐ray crystallography, whereas that of the proximal section is largely unknown [13]. Fibers are thought to be used by the phage for target recognition and anchorage to the host‐cell lipopolysaccharides (LPS) prior to and during DNA translocation [7, 14]. It is so far unknown whether it is the fibers or the tail tube that initially binds to the host cell. Tail fibers have been shown to be in two distinct conformations depending on the infection state [7]. Once new phage progeny are formed and released from the host cell after its lysis, they freely diffuse in search of new host cells [15]. In this freely diffusing viral state, the fibers are in a capsid‐bound, laid‐back position [7, 16]. It is hypothesized that this is a structurally secure conformation, as the fibers are less likely to be accidentally broken off by random collisions with their surroundings. Following target recognition, the phage extends its fibers away from the capsid and toward the bacterium by a currently unknown mechanism. During infection, fibers are in this extended state, with ∼90° between the proximal and distal segments. This angle, however, was calculated from averaged cryo‐electron tomography reconstructions, potentially obscuring the conformational variability of individual fibers [7]. The inability to clearly average the distal regions of the fibers in three‐dimensional reconstructions led to the proposal that the fibers are structurally flexible [11]. We have recently found that T7 phage particles can bind reversibly to the host cell surface and display diffusive host‐surface exploration [16]. Such spatial exploration has also been observed in the case of other virus species, for example, coronaviruses [17]. While the exploration may entail virion rolling or walking, the exact mechanisms and how tail fibers contribute to the process remain unknown.

Here, we explored the structure and large‐scale dynamics of the T7 bacteriophage fiber. We find that fibers display a highly dynamic conformational flexibility along the kink region on the sub‐second timescale. The results indicate that fiber extension is dictated by winding‐unwinding in the coiled‐coil proximal section. The large conformational dynamics and flexibility uncovered in the T7 tail fiber may allow for a walking mechanism during the phage's exploration of the host cell surface and for properly orienting the capsid prior to DNA ejection.

## 2 Results and Discussion

The mechanisms of viral host‐cell recognition and infection processes have been the subject of extensive investigation ever since the discovery of viruses [18]. A special class of viruses, bacteriophages, infect bacteria with high fidelity, hence they emerged as possible agents in specifically targeted anti‐bacterial therapy. However, the exact mechanisms whereby phages recognize and infect their host are still not fully known. Single‐molecule biophysics experiments revealed hitherto unknown, hidden, and highly evolved complexities of the bacteriophage infection cycle, which pointed to the importance of structural dynamics and mechanics in the process [19]. Recent advances in single‐particle biophysics [2], particularly in high‐resolution, time‐resolved atomic force microscopy (AFM), offer the possibility of gaining unique insights into the molecular‐scale mechanisms of viral processes in the native state [20, 21]. In the present work, we investigated the structural dynamics of the tail fibers of the T7 bacteriophage, which are hypothesized to play an important role during the host recognition process [12].

### 2.1 In Situ and Isolated Tail Fibers Display a Similar Global Structure

**FIGURE 1:**
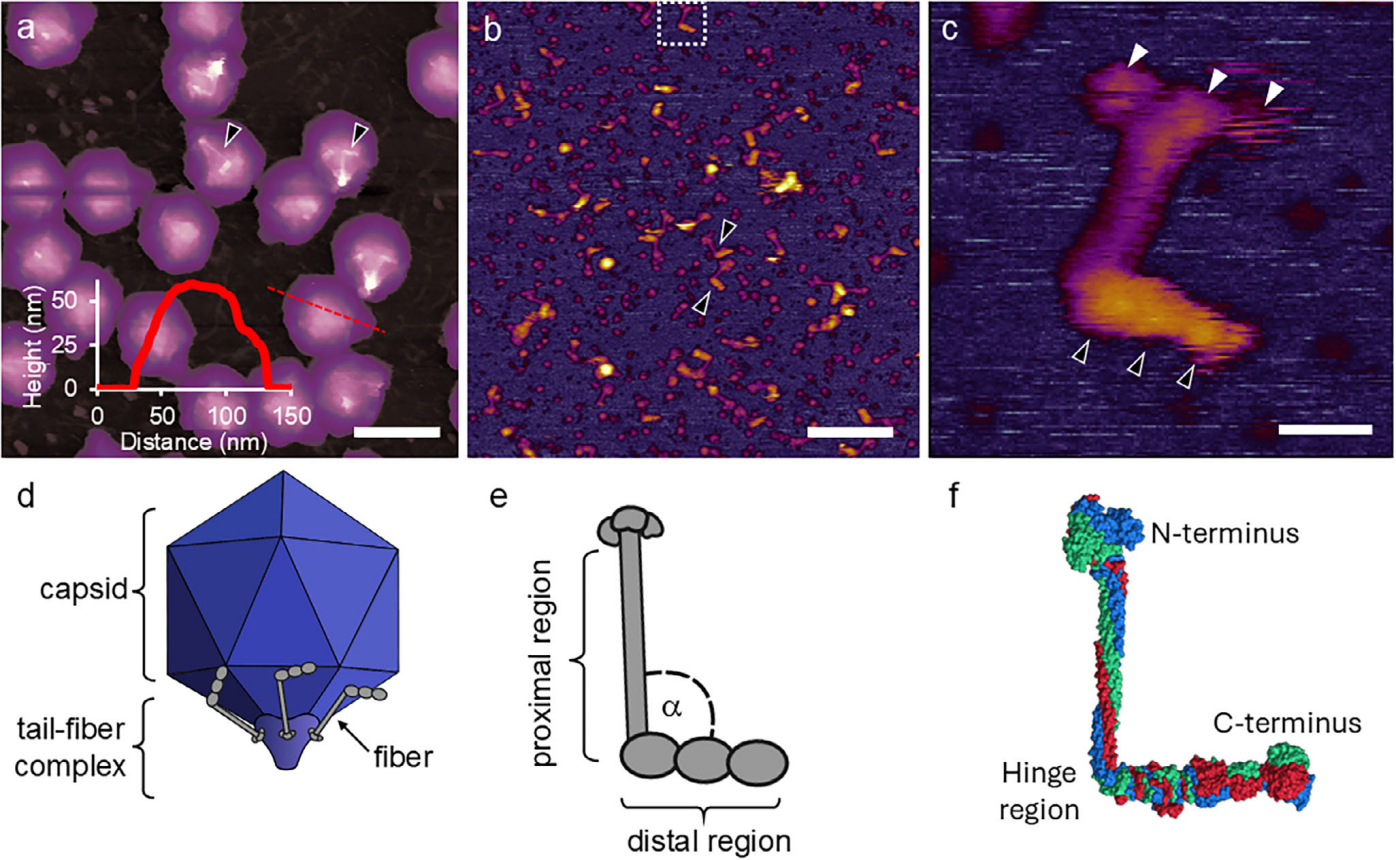
Topographical structure of T7 tail fibers. (a) Height‐contrast AFM image of intact T7 bacteriophages. Capsids and tail fibers are displayed in purple and white colors, respectively. Black arrowheads point at virus‐attached tail fibers. The gray horizontal stroke is an unfortunate imaging artefact. Inset shows the height section profile of a single phage along the white dashed line. Scale bar 100 nm. (b) Height‐contrast AFM image of purified, substrate‐adsorbed tail fibers. Black arrowheads point at particularly well‐resolved tail fibers. White dashed square shows a fiber of interest, enlarged in panel (c). Scale bar 100 nm. (c) Image of a single tail fiber in the more frequent, hence more probable, counterclockwise orientation. Black and white arrowheads point at the three distal and three proximal globules, respectively. Scale bar 10 nm. (d) Simplified schematic representation of a T7 bacteriophage with its tail fibers in the laid‐back orientation. (e) Schematic representation of a tail fiber, showing its different sections. (f) Equilibrated conformation of the T7 fiber obtained with MD simulation. Individual gp17 monomers are shown in red, blue and green. Intermediate fiber‐tail structures identified during the MD simulation are shown in Movie S1.

To reveal the structure of the T7 tail fiber in situ, intact T7 bacteriophages were first imaged by AFM (Figure 1a). Phages appeared as ∼60‐nm‐high icosahedral particles constructed of penta‐ and hexameric capsomeres. Depending on the orientation of the virions, L‐shaped tail fibers could be discerned on their surface. Typically, only one or two fibers were fully visible in AFM images. Imaging more than two fibers would only be possible if the tail complex pointed exactly upwards, resting on the icosahedral vertex opposite of the tail complex. This is a highly unstable configuration, and the capsids typically rest on one of their faces. When observed from the direction of the tail tip, the fibers were always in the clockwise orientation. The fibers could be partitioned into proximal and distal sections, which were closer and farther from the tail, respectively. The mean bend angle was 94° ± 11° (AVG ± SD, n = 45). Unlike the extended‐fiber conformation observed during infection [22], we found that several fibers of non‐host bound phages were in a secure, capsid‐bound state. Although several fibers deviated from the fully laid‐back conformation, the fully extended infection‐state arrangement previously observed on host‐bound particles was never detected. Nevertheless, tail fibers are known to play a crucial role in target recognition and in stabilizing the virus on the host‐cell surface during DNA translocation [23]. Fibers in the laid‐back, capsid‐attached state were also observed by *Hu* et al. by using cryo‐electron tomography [7]. Nevertheless, quantitative analysis revealed that each phage displayed at least one fiber that was not in the fully laid‐back conformation (with ∼50% having one fiber deviating from the fully laid‐back conformation and the remainder having multiple such fibers). T7 phage with laid‐back, virus‐attached tail fibers is schematically shown in Figure 1d.

To alleviate the technical challenges associated with scanning fibers on the non‐planar surface of the capsid, we investigated the structure of recombinantly expressed tail fibers. AFM images of purified tail fibers on substrate surfaces have also revealed the characteristic L‐shaped structure (Figure 1b). The fibers did not form complexes with each other, owing to the absence of the tail tube complex (gp‐8‐11‐12 proteins). In contrast to virus‐attached fibers, isolated ones were usually in a counterclockwise orientation (93% of analyzed fibers, n = 208, Figure 1c shows a fiber in the counterclockwise orientation). The observation could be explained by the difference in the electrostatic properties of the substrate surfaces: since APTES‐coated mica is positive, whereas the capsid surface is negative [24]. However, considering that the tail fiber surface is predicted by AlphaFold to be largely homogenously positive (Figure S1), the fiber's global orientation is most likely dictated by additional factors such as local charge inhomogeneities of the substrate and non‐electrostatic interactions. The lengths of the recombinant tail fiber's proximal and distal sections were 24.0 ± 2.8 nm and 15.4 ± 2.6 nm (AVG ± SD, n = 208), respectively, which agrees well with prior observations [12]. The proximal section is thinner (∼4 nm) than the distal (∼5 nm). The schematic structure of the fiber is shown in Figure 1e. The proximal end of the fiber often appeared as a group of three separate globules, which are the N‐termini of the three gp17 monomers, normally connected to the phage's tail tube (gp8‐11‐12 complex, Figure 1c). The three globules were usually resolved, but sometimes they merged into a single large globule. The proximal section appeared as a straight, rod‐like region, even though modeling has already suggested that it might contain a triple helical structure formed by the three monomers of gp17 (Figure 1f). The thicker distal section of the fiber is formed by three globular parts in tandem, resolvable in the acquired images. It was already apparent from the AFM images that the angle enclosed by the proximal and distal sections can vary from fiber to fiber, ranging from 63° to 153° (94° ± 7°, AVG ± SD, n = 208, cumulative distribution shown in Figure 4d). To explore the reasons behind the broad angle distribution, AlphaFold modeling, molecular dynamics (MD) simulation, and small angle X‐ray scattering (SAXS) measurements were carried out.

### 2.2 T7 Tail Fiber has a Flexible Hinge

**FIGURE 2:**
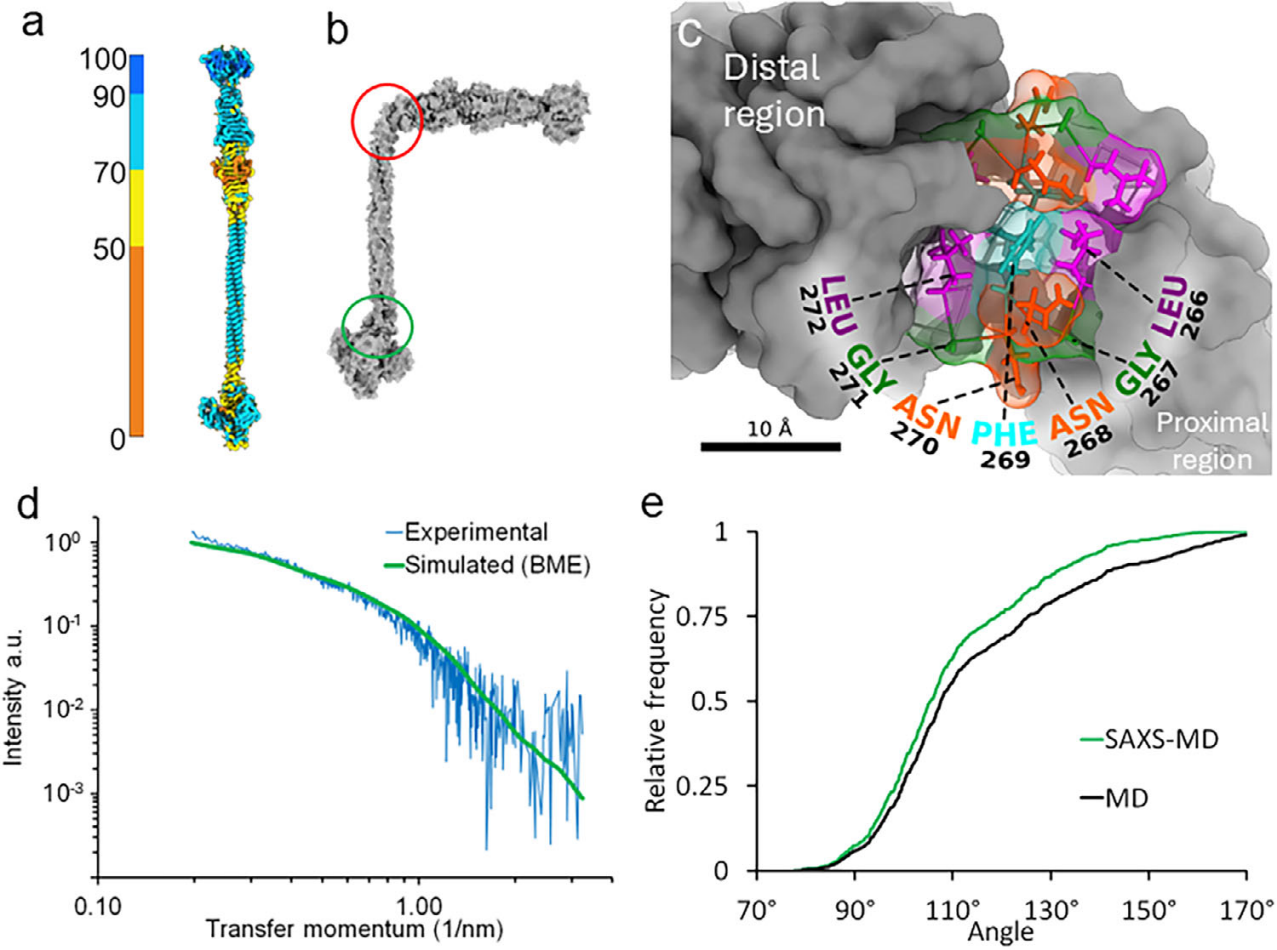
SAXS analysis and MD simulations of the T7 fiber. (a) AlphaFold3‐predicted structure of a gp17 trimer. Color bar indicates the pLDDT score scale. (b) Equilibrium MD‐simulated structure of the gp17 trimer. Green and red circles highlight the shoulder joint (AA: ∼144–160) and kink (AA: ∼266–272) sections, respectively. (c) Structure of the kink section (with all three protein strands displayed). The constituent amino acids in one of the strands are labeled with three‐letter codes. (d) X‐ray scattering results of the gp17 trimer. Experimental data and the BME‐guided fitting curve are shown. (e) Cumulative distribution of the fiber bending angle (see Figure 1e) derived from MD simulation (black) and SAXS‐weighted fitting (green).

The most accurate AlphaFold structure prediction exhibited a straight conformation of the tail fiber (Figure 2a). Predicted local distance difference test (pLDDT) scoring showed high confidence in both the distal and proximal sections. By contrast, the connecting kink region had a much lower confidence score, indicating a potentially dynamic region with less reliable structure prediction. This is in agreement with AFM images showing fibers with a ∼90° bend in this region. The straight structure was used as the initial state for equilibrium all‐atom molecular dynamics (MD). In all cases, the fiber converged toward an L‐shape structure after ∼300 ns (Figure 2b). Analysis of the simulation trajectories identified two flexible regions. One of them is close to the N‐terminus of the trimer; we call this the shoulder joint region (amino acids: ∼144–160). The second flexible region (amino acids: ∼266–272), which is of main interest, is the bent segment separating the proximal and the distal sections. Considering the large angle change in this region, it is a flexible hinge rather than a static kink; hence, we refer to it as the hinge region. During the simulation, most of the conformational changes took place in this hinge region, which led to the ultimate bending of the fiber upon relaxation (Figure 2c). The bent shape appears to represent the most stable conformation, as confirmed by AFM scans and longer (>300 ns) simulations. The energy landscape of the dynamic truncated kink region was calculated, the elastic energy stored in the most likely bent conformations was <10 kT (Figure S2), which means that under equilibrium conditions, the hinge allows for soft conformational transitions. Fiber bend angles from simulated structures were calculated to be 114° ± 21° (AVG ± SD) (Figure 2e). The torsion of the proximal section had a small standard deviation (11°) throughout the simulations.

To experimentally measure the bend angle distribution of the fibers in a non‐immobilized state, we performed small‐angle X‐ray scattering (SAXS), which uncovers conformational ensembles of dynamic proteins, particularly when combined with all‐atom or coarse‐grained MD simulations. The experimental SAXS profile is shown in Figure 2d, blue line. The scattering curve displayed a slight upward turn in the low‐*q* region, suggesting the presence of a small fraction of aggregates. Primary data evaluation followed the guidelines of Jacques et al., and the resulting parameters are summarized in the Supplementary [25]. Notably, the molar mass derived from the forward scattering (182 kDa) agreed well with the theoretical value of the gp17 trimer calculated from the amino acid sequence (189 kDa), providing a robust basis for further analysis. The most probable conformations (out of 750 conformations) were identified by using the Bayesian maximum entropy (BME) approach, which assigned appropriate weights to each frame. Based on these representative conformations and their associated probabilities, the cumulative angle distribution was computed (109° ± 13°, AVG ± SD) (Figure 2e). The MD‐derived distribution closely matches the SAXS‐refined distribution, but it exceeds the bend angle distribution obtained from AFM measurements. Possibly, the surface attachment, necessary in the AFM experiments, places a confinement on both the average and the distribution of the fiber bend angle.

### 2.3 The Proximal Tail‐Fiber Section is a Dynamic Coiled‐Coil

**FIGURE 3:**
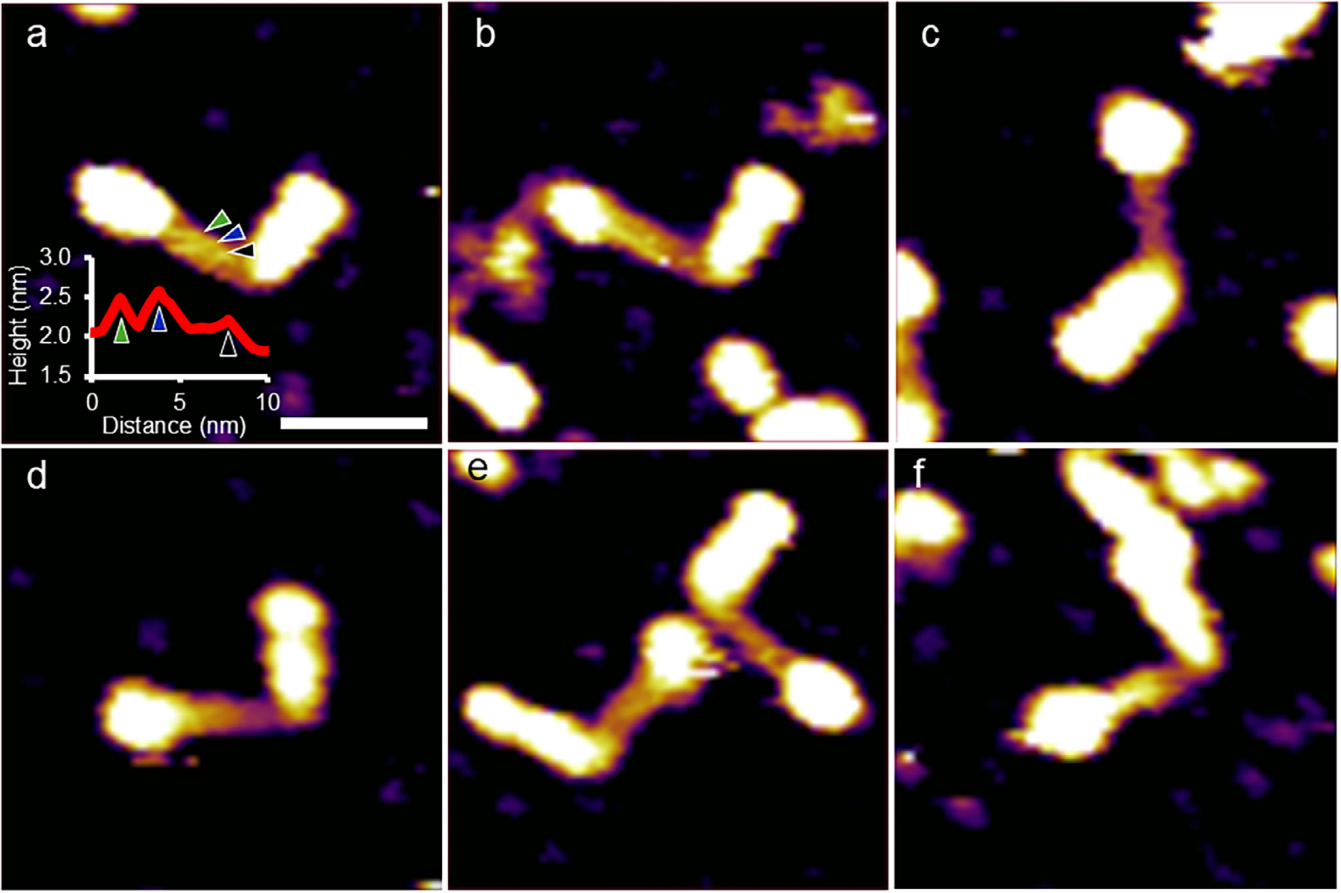
Topographical structure of the T7 fiber's proximal section. Height‐contrast AFM images of isolated, surface‐adsorbed T7 fibers. (a) Fiber with a tightly wound left‐handed triple‐helical proximal section. Inset, height profile along the section's axis. Colored arrowheads mark the strands corresponding to the local height maxima. (b) Fiber with a straightened coiled‐coil section. (c,d) Fibers showcasing different degrees of pitch. (e,f) Fiber proximal regions displaying right‐handed helixes. Scalebar, 20 nm.

AlphaFold predictions and MD simulations both point to the presence of a coiled‐coil region within the proximal section of the fiber. This region has not yet been structurally resolved, likely due to its putative dynamic behavior. To investigate the structure of the proximal section in greater detail, we carried out high‐resolution AFM measurements with a sharp tip (Figure 3). The apparently rod‐like section of the proximal section is, in fact, formed by a triple helical region with a pitch that can vary from fiber to fiber. The three monomers of gp17 can either form a tightly wound helical region (Figure 3a) or they can sometimes be found in an almost straight conformation (Figure 3b), where the three strands run parallel to one another. The whole population of fibers consists of conformational states between these two extremities (Figure 3c–f). Furthermore, interestingly, we observed that the proximal section may acquire either right‐ or left‐handed helical structures, which suggests a large torsional compliance. In a capsid‐connected tail fiber, such torsional compliance likely allows the rotation of the distal section, which may be necessary for fiber extension and host cell surface exploration.

### 2.4 Fiber Bending is Dynamic

**FIGURE 4:**
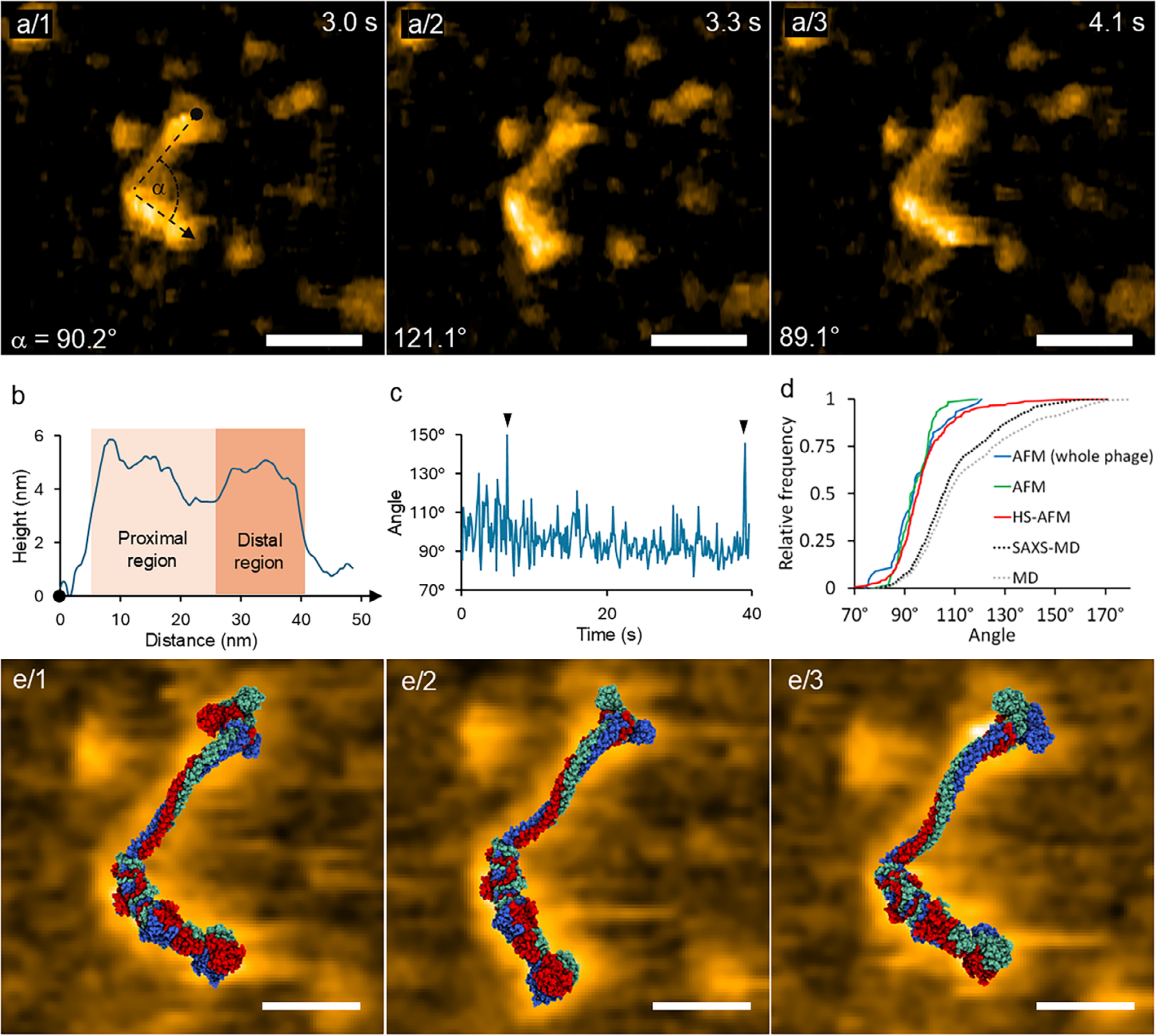
AFM analysis of the T7 fiber kink region. (a) Height‐contrast AFM image series of the same tail fiber (a/1 to a/3). The angles shown refer to the fiber bend angle (α, see Figure 1e), time stamps (top right) indicate the times elapsed from the start of the HS‐AFM recording. Scale bar, 20 nm. Related video, Movie S2. (b) Axial height profile of the tail fiber (along the dashed line in a/1, points and arrowheads corresponding). (c) Fiber bend angle as a function of time. Black arrowheads show extreme cases of fiber bending. (d) Cumulative distribution of fiber bend angles measured by multiple methods. Blue curve shows fiber bend angles measured in the case of capsid attached, whole phages, all other colors showcase bend angles of isolated fibers. (e) Normal‐mode flexible‐fitting (NMFF) of tail fiber molecular structures to their respective HS‐AFM images shown in a. Torsional compliance is indicated by the different orientations of the N‐ and C‐terminal domains. Scale bar, 10 nm.

The results obtained so far provided either snapshots or ensemble averages of the tail fiber's conformation but failed to yield trajectories of motion. To further investigate the hinge region's flexibility and to gain insights into the movement of individual fibers, high‐speed AFM (HS‐AFM) experiments were carried out. Initially, whole T7 phages were scanned, showing the dynamic bending of laid‐back, capsid‐attached fibers (Figure S3 and Movie S6). However, once again, to alleviate the technical difficulties of scanning on the surface of whole phages, isolated fibers were immobilized on the substrate, then imaged at video rate (Figure 4; Movie S2). The overall structure of the fibers appeared similar to that observed with conventional AFM. At a frame rate of 10 s^−1^, we could observe the movement of the distal arm (Figure 4a). Initially, the fiber shown here had a bend angle close to 90° (Figure 4a/1), then it transferred into a somewhat straightened conformation (Figure 4a/2), followed by a quick transition back to the initial state. Fiber bend angles were observed to change rapidly, within a tenth of a second, from the more frequent, approximately right‐angle conformation to the straightened state (Figure 4c). Bend angles were measured frame‐by‐frame from HS‐AFM videos (n = 11), out of which the ones with longer observation times were selected (t > 20 s). Bend angle data were then pooled, the cumulative distribution of which (97° ± 13°, AVG ± SD) is shown in Figure 4d. The bend‐angle distributions obtained in the three different AFM measurements (conventional AFM on in situ and purified fibers, HS‐AFM) are similar; although the angles vary between 70° and 180°, the majority of the data is distributed narrowly around ∼95°. By contrast, in bulk solution (MD and SAXS‐MD measurements), the bend angles are distributed more broadly, and their mean is shifted to a greater value (∼111°). Plausibly, association with the surface kinetically traps the fiber in a conformation with a smaller bend angle. Notably, however, this is the in situ tail‐fiber conformation in the freely‐diffusing, pre‐infection state of the T7 phage. Thus, the in situ conformation is achieved by structural restriction of an otherwise dynamic, torsionally compliant, and bendable tail fiber. Finally, we note that the application of normal‐mode flexible‐fitting (NMFF) to the tip‐convolution‐limited HS‐AFM images with all‐atom‐simulated fiber‐tail structures confirmed both the bending and the torsional dynamics (Figure 4e). Torsion was most apparent in the N‐terminal region; however, torsion/twisting could be observed all along the fiber structure (proximal and distal sections).

**FIGURE 5:**
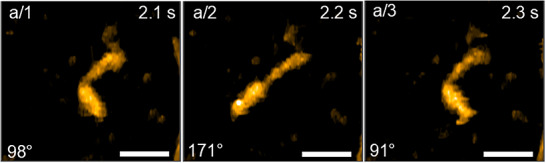
Complete tail fiber straightening. a Height‐contrast AFM image series of the same tail fiber (a/1 to a/3). The angles shown refer to the fiber bend angle (α, see Figure 1e), time stamps (top right) indicate the times elapsed from the start of the HS‐AFM recording. Scale bar, 20 nm. Related HS‐AFM video, Movie S3. NMFF shown in Figure S4.

In rare cases, fibers could even change into a completely straightened conformation within less than 0.1 s, then quickly jump back to ∼90° with the same speed (Figure 5; Movie S3). Notably, the magnitude of the angle change resembles the results of the MD simulation, even though the time scales are different.

We have previously recorded the movement of T7 bacteriophage particles on *E. coli* surface, directly revealing the process of viral host surface exploration [16]. The underlying molecular mechanisms, however, remained hidden. The flexible hinge region of the fiber, characterized here, provides conformational freedom allowing for viral host exploration by a molecular walking mechanism (Figure 8). The collective (i.e., six fibers per virion) dynamics of bending‐straightening modulated by the kinetics of fiber‐LPS binding‐unbinding may result in virus walking, which is manifested in a two‐dimensional diffusive search of the T7 virion across the *E. coli* surface. The phage particles are predicted to explore an area greater than that suggested by the bend angles measured with HS‐AFM, because, as the SAXS‐MD results indicate (Figure 4d), bend angles cover a much wider distribution if the fiber is not surface immobilized. It is likely that the flexible region at the N‐terminal globular end of the tail also contributes to the walking process with a yet‐to‐be explored molecular mechanism.

### 2.5 The Proximal Fiber Section Can Unwind

**FIGURE 6:**
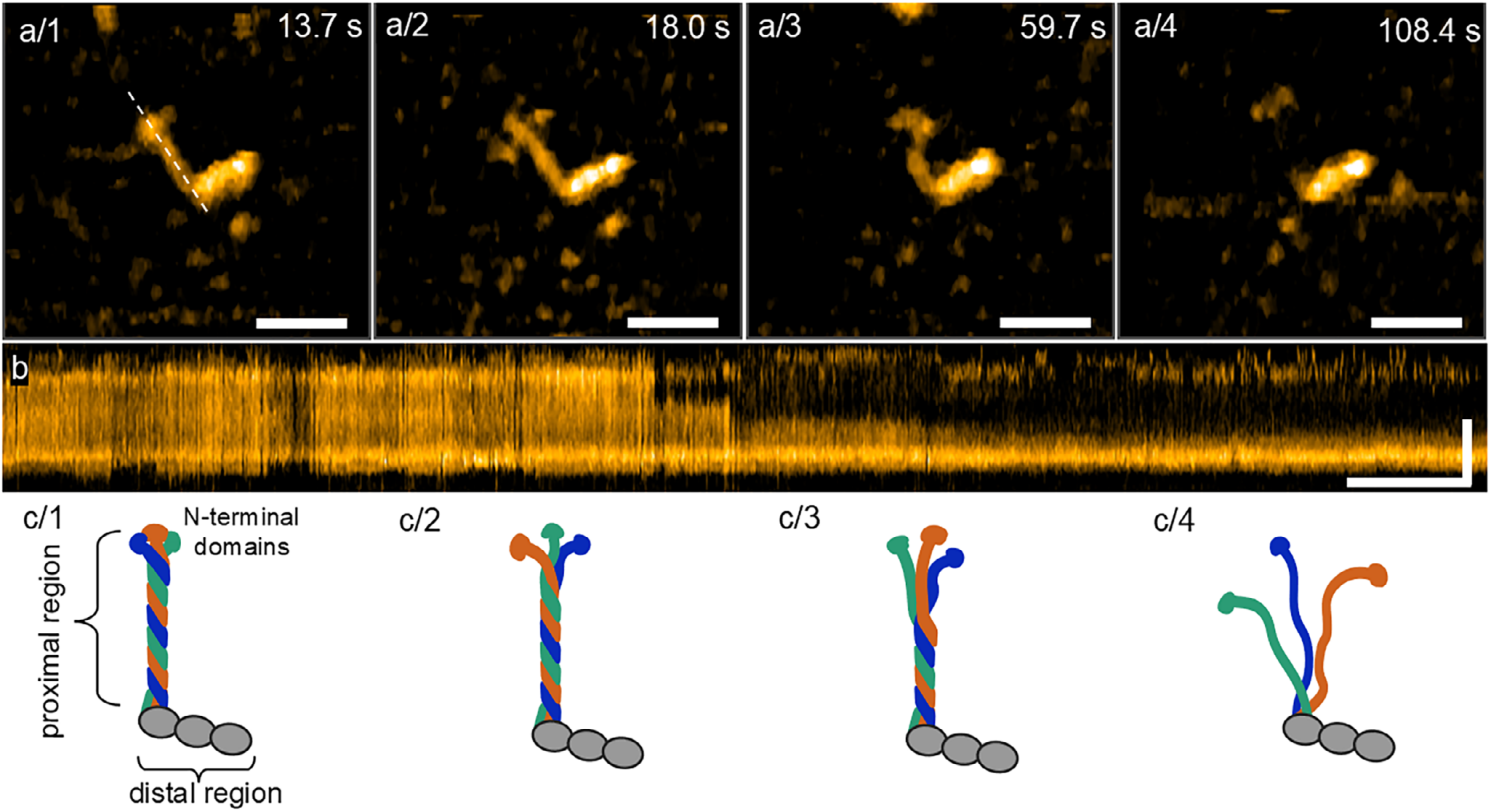
Progressive proximal section unwinding. (a) Height‐contrast AFM image series of the same tail fiber. a/1 shows the starting, folded triple‐helical proximal section, which becomes completely unfolded in ∼1.5 min (a/4). White dashed line in a/1 indicates the path along which a kymogram was collected (displayed in b). Scale bar, 20 nm. (b) Kymogram of the proximal section's unfolding, which was constructed by the line‐by‐line horizontal combination of pixel data from each frame of Movie S4 along the white dashed line in a/1. Scale bars are 10 s and 10 nm in the horizontal and vertical directions, respectively. (c) Schematic images representing the probable fiber structures corresponding to the respective panels in a/1‐4. Related HS‐AFM video, Movie S4.

The conventional AFM images have already suggested that the helicity of the fiber's proximal coiled‐coil region may be altered, hence the three globules at the N‐terminal end may be rearranged. To uncover the dynamics of the process, we carried out HS‐AFM measurements, which revealed that the N‐terminal globules and the coiled‐coil region can indeed rearrange and unwind, respectively, on the 1 min timescale (Figure 6a; Movie S4). The N‐terminal end of the proximal section shown here appeared initially as a single 6‐nm‐diameter globule (Figure 6a/1). Within a few seconds, the N‐terminal globule dissociated, and three smaller globules became visible (Figure 6a/2). These globules are most likely the ends of the gp17 monomers, which are normally bound to the tail‐tube complex (gp8‐11‐12). The three globules wiggled around, presumably rotating around each other, leading to the partial (Figure 6a/3) and finally complete unfolding of the triple‐helical coiled‐coil region (Figure 6a/4). The unwinding process halted at the hinge region, hence the distal section's structural integrity remained unaffected. Kymographic analysis of the proximal section reveals the dynamics of unwinding, and schematics depict the plausible explanation of the process (Figure 6b,c). Furthermore, we note that in situ the N‐terminal end of the fiber is anchored in the tail‐tube complex between the gp11 dodecamer and the gp12 hexamer [11], which may provide structural stabilization and cast a limit on the magnitude of proximal region unwinding, hence to global fiber rotation/twisting. Conversely, fiber rotation may in principle induce a relative rotation between the gp11 dodecamer and gp12 hexamer rings, thereby playing a role in triggering DNA ejection.

### 2.6 Fibers of Tail‐Fiber Complexes can Bend and Twist

**FIGURE 7:**
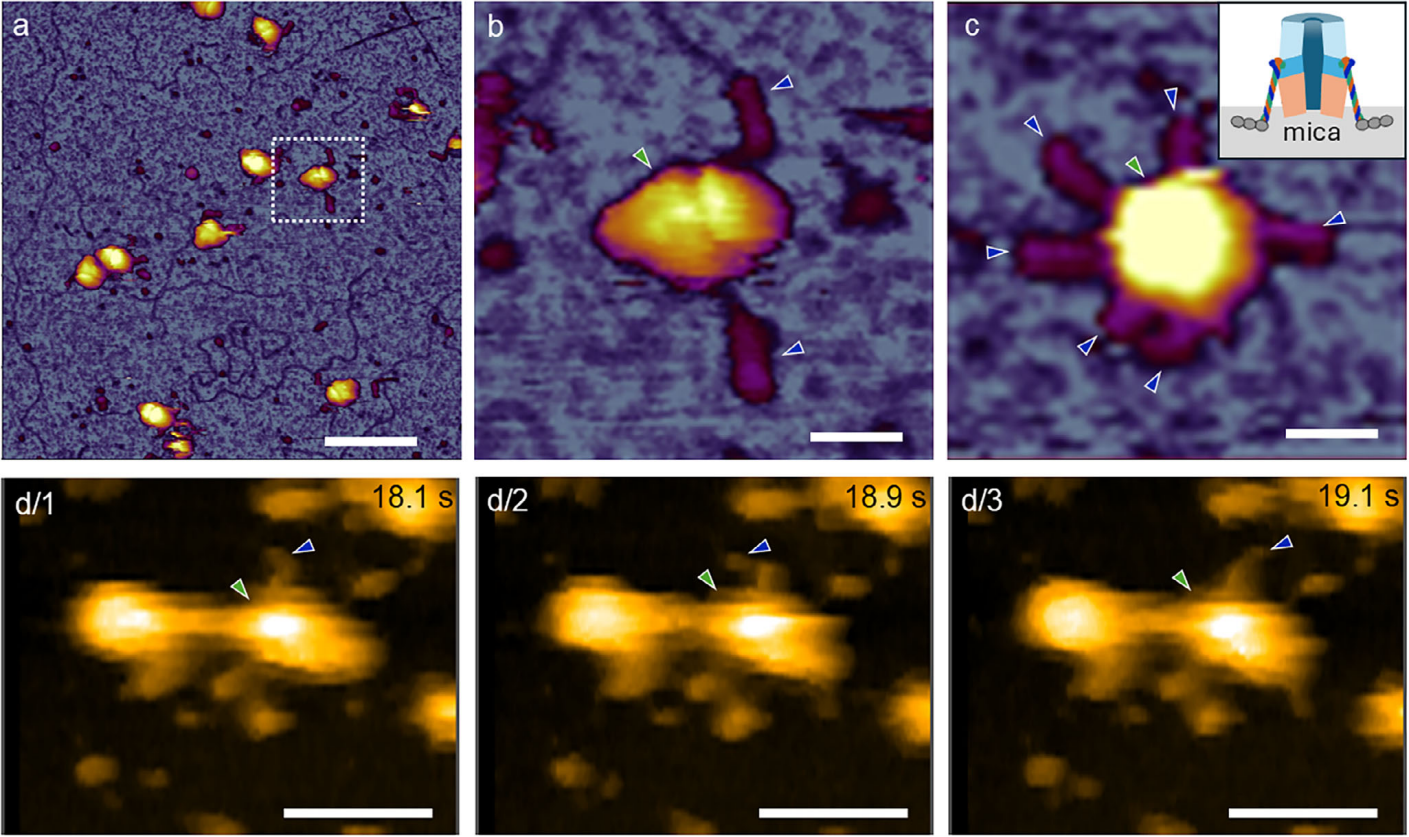
Tail tube associated fibers bend and twist. (a) Height‐contrast AFM image of tail tubes and tail‐fiber complexes. White dashed square shows a region of interest enlarged in (b). Scalebar is 100 nm. (b) Height‐contrast image of a tail‐fiber complex with two visible tail fibers. Scalebar is 20 nm. Blue arrowheads point at fiber distal segments, green arrowhead points at the tail tube, here and in all subsequent panels. (c) Image of another tail‐fiber complex with five to six fibers. Scalebar is 20 nm. Inset in top right shows the side view schematics of a tail‐fiber complex in an upright position, such as that in (c). (d) Snapshots of HS‐AFM image series of the same tail‐fiber complex showcasing fiber bending and twisting. d/1 to d/2 shows a bending motion, while the transition from d/2 to d/3 shows a twisting motion. Scalebar is 50 nm. Related HS‐AFM video, Movie S5.

To observe fiber motions in a more native, N‐terminally anchored state, we have expressed and isolated T7 tail tubes (gp8‐11‐12), which were mixed with tail fibers, consequently forming tail‐fiber complexes. AFM scans revealed central conical structures (tail tubes) with a variable number of fibers attached to them (Figure 7a). Some tail‐fiber complexes had fewer (Figure 7b), and some appeared to have all six fibers (Figure 7c). Depending on the number of fibers in a tail‐fiber complex, its orientation on the surface was distinctly different, which led to two different topologies: Tail‐fiber complexes were either found resting on their side (0‐3 fibers, Figure 7b) or facing upward (>5 fibers, Figure 7c). In both cases, mainly the distal segments of the fibers were visible. HS‐AFM imaging of tail‐fiber complexes has revealed fibers bending and twisting, which is in accordance with the observation of isolated fibers (Figure 7d; Movie S5). Fiber motions were clearly visible in the case of tail‐fiber complexes resting on their side, due to the main axis of fibers being parallel to the substrate surface. Bending motions occur on a similar timescale and to the same degree as that observed in the case of isolated fibers (structural transition from Figure 7d/1,d/2). It is clear that bending occurs along the already characterized hinge region, whereas, based on our results, twisting is consistent with the unwinding/rewinding mechanism proposed from the isolated‐fiber measurements (structural transition from Figure 7d/2,d/3). Twisting may be the direct result of rapid transitions between right‐handed and left‐handed triple helix conformations. We propose that similar fiber conformational dynamics could be possible in the case of whole T7 phages.

### 2.7 Fiber Dynamics Facilitate Critical Transitions of T7 Phage Infection

**FIGURE 8:**
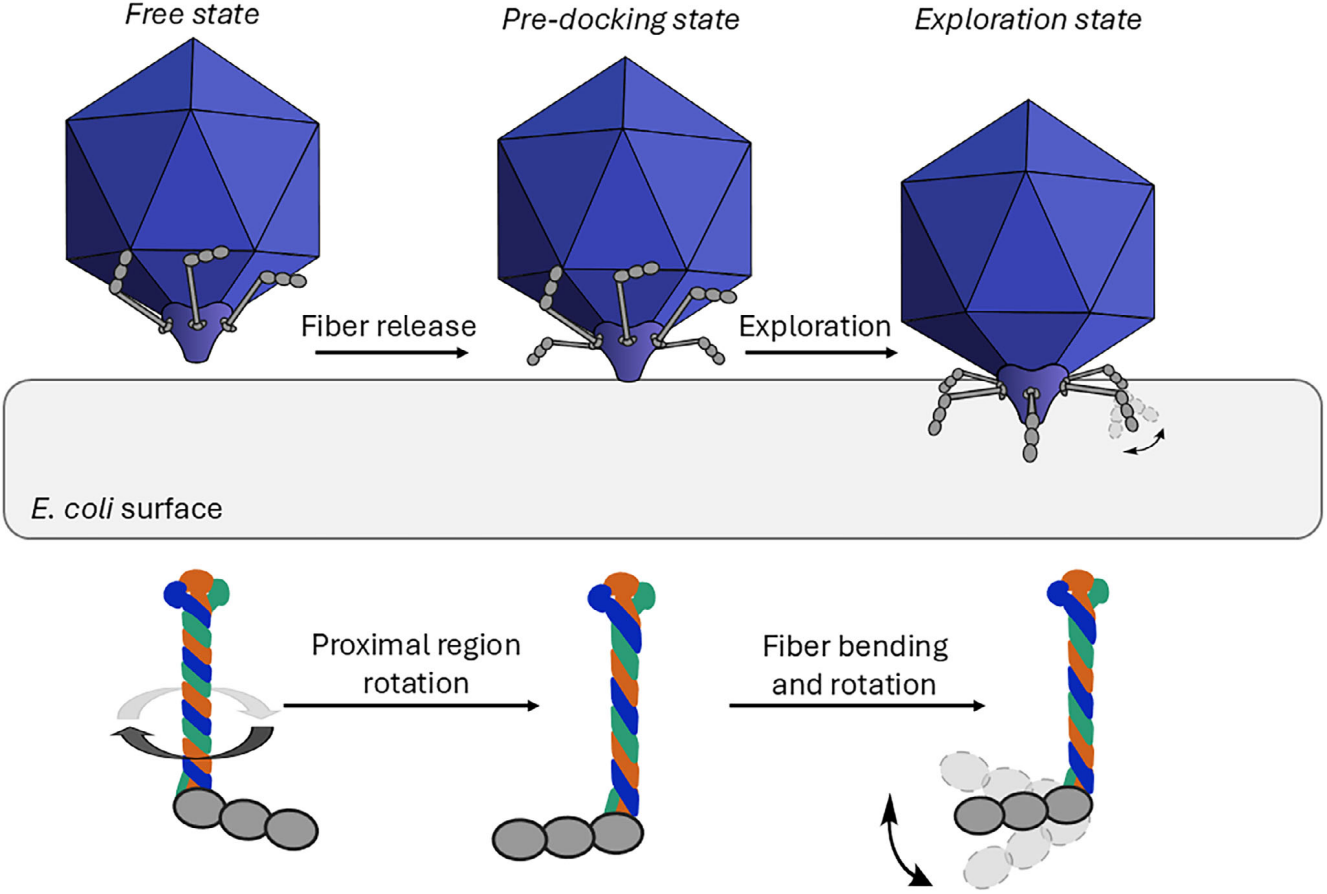
T7 fibers extended by rotation along the proximal section, walking and receptor searching enabled by the presence of a hinge region.

Considering that during infection the T7 tail fibers make a remarkable transition from their fixed‐angle, capsid‐bound state to an extended conformation in which, following a diffusion‐driven search process, all six fibers become properly oriented and attached to surface receptors on *E.coli*, we propose the following empirical model to explain the role of the large‐scale torsional and bending fiber dynamics discovered herein (Figure 8). At present, how the initial fiber‐release event is triggered, and whether it occurs spontaneously or is induced by receptor binding, remains unknown. The T7 tail fiber possesses torsional and bending flexibility and conformational dynamics. The relaxed, lowest‐energy conformation of the fiber is one in which it is bent at an approximately right angle (∼90°–110°) and laid back on the capsid surface. Transitioning from this relaxed state likely requires an input of energy, potentially provided by interaction with the *E. coli* surface. This excited state is characterized by torsional and bending elastic energy stored in the fiber structure. The fate of the elastic energy depends on the coupled kinetics of fiber‐LPS association‐dissociation; that is, by how many fibers are attached to the *E. coli* surface at once. If only one fiber is attached, then the probability of complete T7 particle dissociation from *E. coli* is large, possibly followed by the fiber relaxing back to the virion surface. If a few (but not all) fibers are attached at the same time, then the coupled fiber‐LPS association‐dissociation kinetics allow for the “walking” of the T7 particle on the *E. coli* surface, driven by the concerted, albeit spatially and temporally random, twisting‐bending motion of the involved fibers. The conformational relaxation of one of the fibers allows it to make a step, whereas spatial confinement caused by the other, still attached fibers, increases the probability of this transiently dissociated fiber to re‐bind to the *E. coli* surface, but at a new location. Thus, the large‐scale structural dynamics of the individual fibers together with their host‐surface association‐dissociation kinetics coupled via the tail‐fiber complex may explain the facilitated surface diffusion we have observed earlier [16]. As the number of receptor‐bound fibers increases, the probability of returning to the fully relaxed capsid‐bound state is expected to decrease. Eventually, repeated cycles of fiber attachment and detachment may progressively increase the number of host‐bound fibers until all six fibers become associated with the host surface in the conformationally excited state, making the virion docked and ready for genome transfer [7]. It is an intriguing possibility that the synchronized conformational relaxation of the six fibers contributes not only to orienting the tail properly for DNA ejection, but also to triggering the process by inducing a relative rotation between the gp11 dodecamer and gp12 hexamer rings. A similar trigger mechanism has been proposed in the case of cyanophages, which are members of the T7‐like group of viruses [26]. Even though the observation of fibers and tail‐fiber complexes in an isolated state has certain limitations, such as the lack of host cell surface vicinity, the large‐scale structural dynamics of the T7 tail fiber uncovered in our work may contribute to explaining how the phage transitions from a quiescent state into a deadly infective agent.

## 3 Conclusions

We have shown that the tail fiber of the T7 phage displays large‐scale structural dynamics that drastically distort its global shape on a time scale relevant for infecting its *E. coli* host. These dynamics arise from a flexible hinge region and a torsionally compliant proximal coiled‐coil, which together enable large conformational changes of the fiber. The dynamics involve twisting and bending motions that may collectively give rise to a walking mechanism whereby the virus makes a searching motion on the host surface, indicating that the transition from the freely diffusing to the infective state has a hidden and delicate complexity. The proposed walking mechanism may not be unique to T7 and could potentially be shared by other members of the *Podoviridae* family; however, this possibility requires further investigation. Modulating this process should provide efficient means to target bacteria specifically, hence alleviate the grave problem of antibiotic‐resistance. Finally, the conversion of large‐scale protein conformational changes into surface walking may in principle be utilized in devising novel nanorobotics applications.

## 4 Materials and Methods

Purified T7 bacteriophages were a kind gift from the group of Gabriella Csík [27]. One Shot BL21(DE3) pLysS Chemically Competent *E. coli* (Thermo Scientific) was transfected with gp17‐coding plasmid, which was a kind gift of Mark van Raaij (Centro Nacional de Biotecnología (CNB‐CSIC)). Lysogeny broth (LB), glutaraldehyde grade I (GA) solution, poly‐L‐lysine (PLL) solution, 3‐Aminopropyl triethoxysilane (APTES), IPTG, imidazole, and basic chemicals were purchased from Merck (Darmstadt, Germany). HisPur Ni‐NTA Resin was purchased from ThermoFisher Scientific. Nitrogen 5.0 gas was from Linde Gáz Magyarország Zrt (Budapest, Hungary). Water was purified with a Milli‐Q Integral 3 Water Production Unit (Merck Millipore, Billerica, MA). Round mica sheets were from Ted Pella, Inc. (Redding, CA).

### 4.1 Expression and Isolation of Tail Fibers

Preparations of tail fibers were done according to the work of *Garcia‐Doval* et al. [28]. Briefly, cultures of BL21 *E. coli* were transfected with PET30(+) plasmid encoding the T7 tail fiber (gp17) protein. Transfected bacteria were grown overnight in LB growth medium containing kanamycin (50 µg/ml) and chloramphenicol (34 µg/ml). On the following day, 4 × 250 mL growth media were inoculated with 4 × 2 mL overnight culture and grown while shaking at 37°C to OD_600_ ∼0.6. Expression was induced with 100 µm IPTG, then cultures were grown for 16 h at 16°C while shaking. Cells were collected by centrifugation with 5000 g for 10 min. If necessary, pellets were stored at −80°C. Pellets were resuspended in 30 mL lysis buffer (50 mm TRIS, 4% (v/v) glycerol, 150 mm NaCl, pH 8, supplemented with a protease inhibitor cocktail (Pierce, A32961)). Resuspended cells were lysed by sonication. The lysate was cleared by centrifugation with 38 000 g for 40 min at 4°C. The supernatant was loaded onto a Ni‐NTA column, then, after washing with a buffer containing 50 mm imidazole, the His‐tag labeled fibers were eluted with a 500 mm imidazole buffer. 500 µl fractions were collected and flash frozen in liquid nitrogen, then stored at −80°C. Proteins were stable for at least 3 weeks at 4°C. Samples were further cleaned and concentrated in PBS by using 100 kDa Amicon Ultra centrifugal filters (Merck). Protein concentration of ∼2 mg/ml could be achieved by combining multiple fractions. The presence of gp17 was confirmed by SDS‐PAGE.

### 4.2 Expression, Isolation of Tail Tubes, and Tail‐Fiber Complex Formation

Preparations of tail tubes were done according to the work of Cuervo et al. [10]. C41 *E. coli* cultures were transfected by pRSET‐B plasmid encoding the proteins of the tail tube: gp8, gp11, and gp12. The transfected bacteria were grown overnight in LB growth medium containing ampicillin (100 µg/ml) and chloramphenicol (34 µg/ml). On the following day, 4 × 250 mL growth media were inoculated with 4 × 3 mL overnight culture and grown while shaking at 37°C to OD_600_ ∼0.6. Expression was induced with 100 µm IPTG, then cultures were grown for 3 h at 37°C while shaking. Cell collection and protein purification steps were the exact same as described above for tail fibers. Protein concentration of ∼1 mg/ml could be achieved by combining multiple fractions. The presence of tail tube proteins was confirmed by SDS‐PAGE. 500 µl fractions were collected and flash frozen in liquid nitrogen, then stored at −80°C. Tail‐fiber complexes were formed by mixing tail tubes with tail fibers in a ∼1:6 molar ratio, followed by incubation at 30°C for 1 h, and stored at 4°C. Tail‐fiber complex formation was confirmed by AFM imaging.

### 4.3 AFM Experiments

#### 4.3.1 Imaging of Whole T7 Bacteriophages

Surfaces for Atomic Force Microscopy (AFM) imaging were prepared by dropping 100 µl of PLL onto freshly cleaved mica, followed by 10 min incubation, then washing with deionized water and drying with a stream of high‐purity nitrogen. Then, 100 µl of 25% (w/w) GA was dropped on top of the PLL‐coated surface, which was incubated for 20 min, washed, and dried. 100 µl of 100–200x diluted T7 bacteriophages (∼10^11^ PFU/ml) was applied onto the PLL‐GA coated mica and incubated for 30 min. Unbound phages were washed away by repeatedly pipetting and removing 100 µl of PBS. All the above steps were performed at room temperature. Imaging was performed in PBS (137 mm NaCl, 2.7 mm KCl, 8 mm Na_2_HPO_4_, 1.5 mm KH_2_PO_4_) (pH 7.2) with an Oxford Instruments, Cypher ES scanner, using an Olympus BLAC‐40TS cantilevers (typical spring constant of 90 pN/nm) in non‐contact mode, with scanning speeds of ∼1 µm/s. Imaging was carried out at 25°C.

#### 4.3.2 Imaging Tail Fibers

AFM surfaces were prepared by dropping 100 µl of 10 000 x diluted APTES onto freshly cleaved mica, followed by washing with deionized water and drying in a nitrogen stream. AFM experiments were conducted by dropping 100 µl of 1000–10 000 x diluted fiber samples (∼1–10 µg/ml) onto the APTES‐coated mica. Samples were incubated at room temperature for 5 min, then washed 5 x with PBS. Imaging was performed as described above.

#### 4.3.3 High Resolution Imaging of Tail Fibers and Tail‐Fiber Complexes

100 µl of 25% (w/w) GA was dropped on top of the APTES‐coated surface and incubated for 20 min, followed by washing with deionized water, then drying in N_2_ stream. Fibers were incubated on this surface and washed as described above. Imaging was carried out in PBS, with a Bruker PEAKFORCE‐HIRS‐F‐B cantilever (nominal tip radius ∼1 nm) in non‐contact mode by using a Cypher ES scanner. The sample holding area was flooded with ∼3 mL PBS, then closed and set up was left to equilibrate for 1 h at 20°C.

Samples of surface‐immobilized tail‐fiber complexes were prepared in the same manner, but imaging was done by using Olympus BLAC‐40TS cantilevers in non‐contact mode, with scanning speeds of ∼1 µm/s.

### 4.4 HS‐AFM Experiments

#### 4.4.1 Imaging Tail Fibers and Tail‐Fiber Complexes

High‐speed AFM (HS‐AFM) substrate surfaces were prepared in a manner similar to those in conventional AFM experiments; however, imaging was performed in 3 x diluted PBS to optimize the degree of sample‐to‐surface binding, and a small mica disk was mounted on top of a matching glass stage, which was directly glued onto the Z‐piezo scanner. The sample stage was immersed in a cell containing ∼50 µl of 3 x diluted PBS. HS‐AFM imaging was performed in non‐contact mode by using a laboratory‐built apparatus (WPI‐NanoLSI, Kanazawa, Japan) [21]. Scanning was carried out with custom‐made Olympus AC10 cantilevers with resonance frequencies of ∼200–600 kHz in water and with a typical spring constant of 100 pN/nm. Cantilevers were oscillated with 6–7 nm amplitudes. Scanning was performed with tip velocities of ∼100 µm/s on an 80 × 80 nm region, thereby achieving ∼10 frames per second. 2 × 2 pixel median filtering was applied to the images to improve signal‐to‐noise ratio.

### 4.5 AFM Data Analysis

Images acquired with Cypher ES were post‐processed and analyzed with the AFM driving software (AR 16 running under Igor Pro 6, Wavemetrics, Lake Oswego, OR). HS‐AFM data was post‐processed and analyzed with the UMEX viewer developed by NanoLSI (Kanazawa, Japan). Fibers were detected and analyzed by using a custom in‐house algorithm. Resulting data were sorted, processed, and finally plotted with Origin and Microsoft Excel.

### 4.6 Application of Flexible Fitting to HS‐AFM Images

The molecular model obtained from MD simulations was used in flexible fitting applications to infer atomistic‐precision models of dynamic conformations from HS‐AFM topographic images. We employed the normal‐mode flexible fitting AFM (NMFF‐AFM) method implemented in the BioAFMviewer software [29, 30, 31]. The raw HS‐AFM topographic images for fitting were pre‐processed by applying automatic tilt correction of the AFM stage and a Gaussian filter with 0.5 nm standard deviation. The tip shape parameters to generate simulation AFM images during the fitting process were R = 1 nm for the probe sphere radius and ϑ  =  10° for the cone half angle. Flexible fitting was performed with the following set of parameters: elastic network cutoff distance 8 Å, number of normal modes 15, RMSD of structural deformation 0.5 Å, number of iterations 200–300, image correlation coefficient for image similarity scoring.

### 4.7 SAXS Experiments and Data Analysis

SAXS measurements were performed at the Austrian SAXS beamline of the Elettra synchrotron (Trieste, Italy) in transmission geometry [32]. Measurements were conducted with the 8 keV X‐ray energy branch of the monochromator. Using a Pilatus3 1 M two‐dimensional position sensitive CMOS hybrid pixel detector (Dectris Ltd, Baden, Switzerland), the scattering patterns were recorded in the range of 0.1–5 nm^−1^ in terms of the scattering vector *q* (defined as $q=\frac{4\pi }{\lambda }\;\sin\left(\theta \right)$, where 2θ is the scattering angle and λ is the X‐ray wavelength). To be able to assess sample stability during the experiment, twenty exposures, each 10 s long, were made and repeated for each sample. The relevant aqueous medium was measured before and after the measurement series. The corrected scattering patterns were azimuthally averaged to yield one‐dimensional scattering curves for each sample using SAXSDOG [33]. The individual exposures were corrected for beam flux, geometric effects, and for sample transmission, and the µ‐drop autosampler installed at the beamline [33]. The background scattering curve was subtracted in the case of the measurements of fibers. The corrected scattering patterns were azimuthally averaged to yield one‐dimensional scattering curves for each sample.

### 4.8 Molecular Dynamics Simulation and Data Analysis

Structural prediction of the gp17 (Uniprot ACC: H6BFI5) homotrimer protein complex was carried out by using the AlphaFold Server [34]. The multimer with the highest ranking‐score (0.61) was used for subsequent simulation with a disordered fraction of 0.26, pTM of 0.5, and ipTM of 0.48.

All‐atom molecular dynamics (MD) simulations were performed using GROMACS 2023.2 with the CHARMM27 force field [35]. The initial protein structure was obtained from AlphaFold predictions and placed in a rectangular simulation box with edge lengths of 30, 35, and 20 nm. The system was solvated using the TIP3P water model. Sodium (Na^+^) and chloride (Cl^−^) ions were added to neutralize the net charge. Energy minimization was carried out using the steepest descent algorithm for 5000 steps. Equilibration was performed for 500 ps using a 2 fs integration time step. Long‐range electrostatic interactions were computed using the reaction‐field method with a cutoff distance of 1.2 nm, and van der Waals interactions were treated with the same cutoff [36]. Temperature was maintained at 310.15 K by using the velocity‐rescaling thermostat with a coupling time of 1 ps, and pressure was isotropically controlled using the C‐rescale barostat with a 5 ps coupling constant [37]. All covalent bonds involving hydrogen atoms were constrained using the LINCS algorithm [38]. Three independent MD simulation runs of 500 ns each were carried out with initial velocities assigned from a Maxwell–Boltzmann distribution using different random seeds. 250 equally spaced frames per trajectory were extracted, yielding a total of 750 conformations.

Theoretical SAXS profiles for each MD frame were computed using CRYSOL 3 [39]. To estimate the optimal constant background level and scaling factor, CRYSOL was first run in fitting mode using the experimental SAXS curve for each structure. From each resulting file, the optimal background and scaling factors were parsed automatically. A second round of CRYSOL was then run without the experimental data, but using the extracted background and scale values, ensuring the resulting intensity files represented unbiased theoretical predictions adjusted for contrast matching. This step was parallelized for several hundred frames using a Python script. To reconcile the conformational ensemble with the experimental SAXS data, Bayesian Maximum Entropy (BME) reweighting was performed [40]. For each frame, the CRYSOL‐generated intensity profile was interpolated to match the experimental q‐range. The BME algorithm was then applied to compute an optimal set of statistical weights w_i_ for the frames by minimizing the Kullback–Leibler divergence D_KL_ from a uniform prior subject to agreement with the SAXS data within a confidence parameter θ. The effective ensemble was defined as:

$$\;\langle I_{calc}\left(q\right)\rangle =\sum_{i}w_{i}I_{i}\left(q\right)$$

The weights were bootstrapped to estimate standard deviations. Frames with nonzero weights were selected as representative structures contributing to the reweighted ensemble. To visualize structural convergence, the corresponding PDB files were loaded into PyMOL 2.

### 4.9 Measurement of Angles and Torsion in MD Structures

Alpha C atoms of the multimer were extracted from the simulated protein structures using UCSF ChimeraX [41]. Two linear fits were done on the extracted C atoms in Python using scikit‐spatial (v6.8.1, 2019, Andrew Hynes): one fit on residues 160–250 and one fit on residues 280–558, thus excluding the regions where the structure bends (hinge region). Angles were measured between these two linear fits. Torsion was calculated with the help of two planes. The first plane was defined at the end of the fiber by using the 480th serine residues of the three monomers. The second plane was defined with the help of the two linear fits (the two opposite ends and their intersections). Torsion was calculated as the angle between subsequent intersections of the two planes.

## Author Contributions

Luca Elizabet Kosik, Andrea Balogh‐Molnár, Bálint Kiss, Ana Cuervo, and Hedvig Tordai prepared samples. Luca Elizabet Kosik, Miklós Cervenak, and Bálint Kiss performed AFM experiments. Soma Yamamoto, Hiroki Konno, Noriyuki Kodera, and Bálint Kiss designed and performed HS‐AFM experiments. Dominik Sziklai and Bence Fehér performed simulations. Bence Fehér, Negar Rahimi, and Heinz Amenitsch performed SAXS experiments and data analysis. Luca Elizabet Kosik, Bálint Kiss, and Levente Herényi performed AFM data analysis. Holger Flechsig and Romain Amyot performed NMFF fitting. Bálint Kiss and Miklós Kellermayer conceived the project and wrote the manuscript. Miklós Kellermayer secured funding. All authors have read and approved the manuscript.

## Conflicts of Interest

The authors declare no conflicts of interest.

## Supporting information




**Supporting File 1**: smll74269‐sup‐0001‐SuppMat.docx.

**Supporting File 2**: smll74269‐sup‐0002‐MovieS1.mp4.

**Supporting File 3**: smll74269‐sup‐0003‐MovieS2.mp4.

**Supporting File 4**: smll74269‐sup‐0004‐MovieS3.mp4.

**Supporting File 5**: smll74269‐sup‐0005‐MovieS4.mp4.

**Supporting File 6**: smll74269‐sup‐0006‐MovieS5.wmv.

**Supporting File 7**: smll74269‐sup‐0007‐MovieS6.avi.

## Data Availability

The data that support the findings of this study are available from the corresponding author upon reasonable request.
