# Supplementary material for: Large‐Scale Structural Dynamics in the Tail Fiber Modulate the Infective Transition of the T7 Bacteriophage
Source: Small. 2026 Jun 19;22(46):e74269. doi: 10.1002/smll.74269 (PMC13485008; doi:10.1002/smll.74269)
Supplement: Supplementary file 1 [file SMLL-22-e74269-s006.docx]

**Supporting information:**

**Large-scale structural dynamics in the tail fiber modulate the infective transition of the T7 bacteriophage**

Luca Elizabet Kosik^1,2^, Miklós Cervenak^1^, Dominik Sziklai^1^, Andrea Balogh-Molnár^1^, Negar Rahimi^1,3^, Bence Fehér^1,3^, Soma Yamamoto^4,5^, Hiroki Konno^6^, Noriyuki Kodera^6^, Holger Flechsig^6^, Romain Amyot^6^, Heinz Amenitsch^7^, Hedvig Tordai^1^, Levente Herényi^1^, Ana Cuervo^8^, Miklós Kellermayer^1,2*^, Bálint Kiss^1,2*^

1. Department of Biophysics and Radiation Biology, Semmelweis University, Tuzolto Str. 37-47, H1094 Budapest, Hungary
2. HUN-REN-SE Biophysical Virology Group, Tuzolto Str. 37-47, H1094 Budapest, Hungary
3. Nanobiophysics Research Group, HUN-REN Office for Supported Research Groups, Budapest, 1094, Hungary
4. Graduate School of Natural Science and Technology, Kanazawa University, Kanazawa, Ishikawa 920-1192, Japan
5. WISE Program for Nano-Precision Medicine, Science, and Technology, Kanazawa University, Kanazawa, Ishikawa 920-1192, Japan
6. WPI Nano Life Science Institute (WPI-NanoLSI), Kanazawa University, Kanazawa, Ishikawa 920-1192, Japan
7. Institute of Inorganic Chemistry, Graz University of Technology, Stremayrgasse 9/V, 8010 Graz, Austria
8. Centro Nacional de Biotecnología (CNB-CSIC), Darwin 3, Cantoblanco, 28049 Madrid, Spain

*Corresponding authors:

[kellermayer.miklos@semmelweis.hu](mailto:kellermayer.miklos@semmelweis.hu)

[kiss.balint@semmelweis.hu](mailto:kiss.balint@semmelweis.hu)

SAXS Data collection parameters

| Instrument | Austrian SAXS beamline@Elettra |
| --- | --- |
| Beam geometry | 0.5 x 1.5 mm transmission |
| Wavelength ( Angström) | 1.54 |
| q range (1/Angström) | 4.7 * 10^-2^ – 3.2 |
| Exposure time (s) | 12x10 s |
| Concentration (mg / mL) | 0.5 |
| Temperature (K) | 303 |

Structural parameters

| I(0) (cm-1) [from P(r)] | 0.037 $\pm$ 0.0023 |
| --- | --- |
| Rg (nm) [from P(r)] | 4.72 $\pm$ 0.04 |
| I(0) (cm-1) [from Gunier fit] | 0.04 $\pm$ 0.0017 |
| Rg (nm) [from Gunier fit] | 4.70 $\pm$ 0.04 |
| Dmax (nm) | 14.43 |
| Porod volume estimate (nm3) | 150 |

Molecular-mass determination

| Calculated from I(0) (kDa) | 182 |
| --- | --- |
| Calculated from sequence (kDa) | 189 |

Software employed

| Primary data reduction | SAXSDog, beamline data pipeline |
| --- | --- |
| Data processing | PRIMUS |
| Guinier analysis | PRIMUS |
| Inverse Fourier transformation | GNOM |
| Molecular dynamics simulation | GROMACS, CHARMM force field |
| BME weighting | Own-written python script |

**Supplementary Fig. 1.** Surface charge of T7 fibers. AlphaFold prediction of fiber surface charge, showing no differences between the two sides of the fiber. Blue color represents positive charge, red represents negatively charged surfaces.

**Supplementary Fig. 2.** Potential of Mean Force (PMF) along the gp17 trimer kink's bending angle estimated by the Accelerated Weight Histogram (AWH) method implemented in Gromacs. PMF is expressed in kT units at 310 K. Sampling time was 1us. AWH sampling was done on the truncated kink region — depicted in the insets — cut out from the full gp17 trimer predicted by AlphaFold3. The trimer remained structurally intact during sampling.

**Supplementary Fig. 3.** HS-AFM image series of fiber bending on the surface of whole bacteriophages, in a raw height-contrast format (top) and a gradient enhanced version (bottom). Snapshots were taken from Movie 6. Please note that the recording has a slight double tip artifact. Samples were prepared in the exact same way as those seen in Fig 1a and described in the methods section “Imaging of whole T7 bacteriophages”, but imaging was done in by HS-AFM .

**Supplementary Fig. 4.** AFM image and NMFF of an extended tail fiber. **a** MS-AFM frame of a fiber in extended conformation. **b** Simulated AFM image of NMFF fitted tail fiber. **c** NMFF structure overlayed on HS-AFM frame shown in **a**. Scalebar 10 nm.

**Supplementary Movie 1:** Structural states of all-atom molecular dynamics simulation of AlphaFold predicted gp17 trimer structure.

**Supplementary Movie 2:** HS-AFM image series showcasing fiber bending.

**Supplementary Movie 3:** HS-AFM image series showcasing quick fiber transitions to an extended (~180° bend angle) conformation.

**Supplementary Movie 4:** HS-AFM image series showing the uncoiling/unfolding of the fiber proximal region.

**Supplementary Movie 5:** HS-AFM image series showing the movement of tail-fibers in case of whole tail complexes.

**Supplementary Movie 6:** HS-AFM image series showing the bending of phage attached fibers in their native state.
